# Rapid Trace Detection of Sulfite Residue in White Wine Using a Multichannel Colorimetric Nanozyme Sensor

**DOI:** 10.3390/foods12193581

**Published:** 2023-09-26

**Authors:** Xiaoyue Yue, Long Fu, Chaoyun Wu, Sheng Xu, Yanhong Bai

**Affiliations:** 1 College of Food and Bioengineering, Zhengzhou University of Light Industry, Zhengzhou 450001, China; 2017059@zzuli.edu.cn (X.Y.); fulong2498394@163.com (L.F.); wuchaoyun0302@163.com (C.W.); 2 Key Laboratory of Cold Chain Food Processing and Safety Control, Ministry of Education, Zhengzhou University of Light Industry, Zhengzhou 450001, China; 3 Henan Key Laboratory of Cold Chain Food Quality and Safety Control, Zhengzhou University of Light Industry, Zhengzhou 450001, China; 4 College of Computer and Communication Engineering, Zhengzhou University of Light Industry, Zhengzhou 450001, China; xusheng@zzuli.edu.cn

**Keywords:** colorimetric detection, multi-channel and rapid detection, nanozyme, sulfite

## Abstract

As a commonly used food additive, sulfite (SO_3_^2−^) is popular with food manufacturers due to the functions of bleaching, sterilizing, and oxidation resistance. However, excess sulfites can pose a threat to human health. Therefore, it is particularly important to achieve rapid and sensitive detection of SO_3_^2−^. Herein, a colorimetric sensor was invented for visual, meticulous, and rapid detection of SO_3_^2−^ based on MIL-53(Fe/Mn). Bimetallic nanozyme MIL-53(Fe/Mn) was prepared by a one-pot hydrothermal reaction. The prepared MIL-53(Fe/Mn) can effectively catalyze the oxidation of colorless TMB to a blue oxidation product (oxTMB). The introduction of SO_3_^2−^ causes significant discoloration of the reaction system, gradually transitioning from a visible blue color to colorless. Hence, a sensitive colorimetric sensor for SO_3_^2−^ detection was developed based on the decolorization degree of the detection system. Further, the discoloration was ascribed to the inactivation of nanozyme and the strong reducing ability of SO_3_^2−^. Under the optimal experimental conditions, there was a good linear relationship between the absorbance at 652 nm and SO_3_^2−^ concentration in the linear range of 0.5–6 μg mL^−1^ with a limit of detection (LOD) of 0.05 μg mL^−1^. The developed method was successfully applied to the detection of actual samples of white wine with good accuracy and recovery. Compared to traditional methods, this colorimetric sensor produces similar detection results but significantly reduces the detection time. Compared to traditional methods, this colorimetric sensor can not only reduce the detection costs effectively but also help the food industry maintain quality standards. Strong anti-interference capability, simple operation, and low detection limits ensure the excellent performance of the colorimetric sensor in detecting SO_3_^2−^ in white wine. The combination of a smartphone and a colorimetric analysis application has also greatly facilitated the semi-quantitative, visual on-site detection of SO_3_^2−^, which has opened up an application prospect of an MIL-53(Fe/Mn)-based detection platform. Our work has indicated a new direction for the detection of SO_3_^2−^ and provided important assurance for food safety.

## 1. Introduction

Due to important functions such as bleaching, decolorization, preserving, and inhibiting browning, sulfite (SO_3_^2−^), as a food additive, is widely used in the food-processing industry [1,2]. However, the excessive addition of SO_3_^2−^ can induce grievous and even life-threatening health problems including polyneuritis, dyspnea, diarrhea, and asthma [3,4,5]. The Joint FAO/WHO Expert Committee on Food Additives has confirmed that the maximum daily intake of nitrite is 0.06 mg kg^−1^ [6]. China also has strict requirements on the amount of sulfites. However, there are many people that add excessive amounts of sulfites in order to make a profit or to meet technical standards, posing a threat to the health of the general public. Therefore, it is essential to develop methods for detection of sulfites in food for food safety and quality assurance. In recent years, sulfite detection technologies have emerged one after another, such as titrimetric iodine [7,8], electrochemical [9,10], chromatographic [11,12], fluorescence [13,14], and colorimetric analytical methods [15,16], among which colorimetric analytical methods are favored by researchers due to their advantages of simple operation, high sensitivity, and low cost. In recent years, with the continuous improvement of imaging capabilities of smartphones, it has become possible to use smartphone-based digital image colorimetry. Recently, there have been many research works that combine smartphones with colorimetry detection, which provide a reliable theoretical basis for the development of this work [17,18,19]. The metal nanoparticle-based colorimetric method is one of the most popular colorimetric assays, however, it relies heavily on the catalytic properties and stability of the nanoparticle itself [20]. There is no doubt that the development of nanozymes with better catalytic performance, stability, and specificity is essential to improve the sensitivity, accuracy, and selectivity of SO_3_^2−^ detection.

Since 2007, when Fe_3_O_4_ was found to possess peroxidase-like mimetic activity, some nanomaterials have been found to possess significant natural enzyme mimetic activity and can effectively catalyze biochemical reactions [21]. As a synthetic nanomaterial, nanozymes have the advantages of being inexpensive, stable, and having adjustable catalytic performance compared with natural enzymes. Moreover, nanozymes are more tolerant to the surrounding environment and are successfully applied in food safety, disease treatment, environmental protection, aerospace, biosensing, and many other fields [22,23]. Currently, there are mainly five types of nanozymes: metal nanoparticle-based nanozymes [24], metal oxide nanomaterial-based nanozymes [25,26], carbon nanomaterial-based nanozymes [27,28], MOF-based nanozymes [29,30], and other types of nanozymes [31,32]. These nanozymes can mimic the catalytic activity of natural enzymes such as peroxidase (OPD) [33,34], oxidase (OXD) [35,36], hydrolase [37], catalase (CAT) [38], glucose oxidase (GOX) [39], and superoxide dismutase (SOD) [40]. Oxidase-like nanozymes are more popular among researchers due to their simpler and more reliable method, as they do not require toxic and decomposable co-substrates such as hydrogen peroxide, compared to peroxidase-like nanozymes.

Metal–organic frameworks (MOFs) are highly ordered network-like porous nanomaterials formed by metal ions or metal clusters and organic ligands bonded to each other through coordination bonds. MOFs have excellent physical and chemical properties such as large specific surface area, versatility, high stability, and adjustable pore size [41,42]. The rapid development of MOF-based material accelerated its wide range of applications in biochemical catalysis, luminescence, gas storage, sensing, adsorption, and separation [43,44,45]. Over decades of rapid development, thousands of nanomimetic enzymes have been introduced, and MOFs based on Fe have high hydrothermal and chemical stability, which are some of the most classic organic frameworks [46]. Although Fe-based MOFs possess many attractive features, they still face many obstacles in practical applications, such as low conductivity and poor electrochemical stability [47]. Likewise, most nanozymes exhibit significant limitations, for example, poor dispersion, low catalytic activity, and stringent operating conditions, which restrict their practical applicability [48]. To address these problems, many groups have created a variety of methods to improve the properties of the nanozyme itself as much as possible, including morphological modifications, heteroatom doping, compounding of multiple nanomaterials, encapsulation, surface modifications, and addition of activators or inhibitors [49,50].

Heteroatom doping is one of the most common means of effectively improving the catalytic efficiency of nanomaterials and can alter the original properties of nanomaterials very well, even to the point of bringing about new and surprising effects [51,52]. For example, Yang’s group successfully prepared CoMo-300r with oxidase-like activity by introducing both Co and Mo through high-temperature calcination. This material was successfully applied for the detection of SO_3_^2−^ in white wine. Compared to CeO_2_, CoMo-300r is rich in oxygen vacancies, exhibiting lower charge transfer resistance and faster interface charge transfer capability, thereby accelerating the kinetics of the redox reactions of the obtained products [53]. In a similar vein, Malakootian et al. achieved precise detection of SO_3_^2−^ in water and soft drinks by doping Ce^3+^ into CuO nanocomposites, with a detection limit as low as 0.08 μM. The method demonstrated high sensitivity, good reproducibility, and stability [54]. In general, the enzyme activity of bimetallic nanozymes is superior to that of monometallic nanozymes [55,56]. The bimetallic coupling synergy can increase the number of reactive sites and facilitate the improvement of catalytic activity, resulting in a qualitative leap in the properties of MOFs. This point has been demonstrated by many bimetallic MOF-derived nanozymes [57]. Inspired by the above, we introduced the element Mn into MIL-53(Fe) to obtain MIL-53(Fe/Mn). In contrast, MIL-53(Fe/Mn) has more excellent catalytic properties.

Herein, we prepared the MIL-53(Fe/Mn) bimetallic nanomaterial by a one-pot hydrothermal technique. Benefiting from the synergy between Fe and Mn elements, the electron transfer rate between MIL-53(Fe/Mn) and TMB was accelerated. Thus, the oxidase simulation ability of MIL-53(Fe/Mn) was significantly increased. It was demonstrated that SO_3_^2−^ could inhibit the mimetic enzymatic ability of MIL-53(Fe/Mn). With the increasing concentration of SO_3_^2−^, the enzymatic activity of MIL-53(Fe/Mn) was gradually weakened, resulting in the decrease in the absorbance of oxTMB. Thus, a colorimetric sensor for SO_3_^2−^ detection was constructed. There is a good linear relationship between the concentration of SO_3_^2−^ and the absorbance of oxTMB. Importantly, the rapid on-site semi-quantitative detection of SO_3_^2−^ was achieved by combining a smartphone with a color recognition application.

## 2. Experimental

### 2.1. Reagents and Materials

FeCl_3_·6H_2_O, Mn(CH_3_COO)_2_·4H_2_O, Na_2_SO_3_, and CH_3_COONa were purchased from Shanghai Macklin Biochemical Co., Ltd. (Shanghai, China). KCl, MgCl_2_, CoCl_2_, anhydrous ethanol, tartaric acid, and other reagents were obtained from Sinopharm Chemical Reagent Co., Ltd. (Shanghai, China). Ion chromatography pretreatment columns were obtained from Ningbo Hongpu Experimental Technology Co., Ltd. (Ningbo, China). White wine was purchased from a local large supermarket chain, and the production date indicated it was fresh. All water used in the experiments was DI water made by the laboratory. The above reagents were stored according to the label requirements, were of analytical purity, and were used directly without any special treatment before use.

### 2.2. Instrumentation

The UV–Vis spectra were measured on a multifunctional microplate detector (TECAN Spark, Grödig, Austria). Scanning electron microscope images were taken on a field emission scanning electron microscope (Regulus 8100, Tokyo, Japan). Transmission electron microscope images were taken by a high-throughput field emission transmission electron microscope (JEOL JEM 2800, Tokyo, Japan). The XPS spectra were captured with an X-ray photoelectron spectrometer (Thermo Scientific ESCALAB 250Xi, Waltham, MA, USA). Infrared spectra were gained from a Fourier transform infrared spectrometer (Bruker, Vertex 70, Saarbrucken, Germany). Electron paramagnetic resonance spectrometry (Bruker E-Scan, Saarbrucken, Germany) was used to trap free radicals. Zeta potentials were obtained by electrochemical workstation (CHI 660e, Shanghai, China) measurements.

### 2.3. Synthesis of Bimetallic Nanozyme MIL-53(Fe/Mn)

MIL-53(Fe/Mn) was prepared by a simple one-pot hydrothermal method that was referred to in a previous report [35]. To be more specific, 0.5404 g FeCl_3_·6H_2_O and 0.9804 g Mn(CH_3_COO)_2_·4H_2_O were dissolved in 20 mL DMF solution, named Solution A. Then, 0.4980 g (3 mmol) of H_2_BDC was dissolved in 20 mL DMF solution, named Solution B. Solutions A and B were stirred and stopped until there were no obvious powder particles to precipitate. Afterwards, solution A was added to solution B and stirred continuously at room temperature for 30 min. Finally, the aforementioned mixture was poured into a Teflon-lined autoclave and heated to 120 °C and held for 8 h. After natural cooling, the light brown precipitate was obtained by centrifugation. Next, the precipitate was shaken with DMF and centrifuged at 9000 rpm/min for 15 min. And then, the precipitate was washed with anhydrous ethanol under the same conditions for three times. Finally, the resulting precipitate was dried overnight at 60 °C and placed under seal at normal temperature. For comparison purposes, the monometallic nanomaterial MIL-53(Fe) was synthesized simultaneously in the same way as the above operation.

### 2.4. Oxidase Activity of MIL-53(Fe/Mn)

The catalytic activity of MIL-53(Fe/Mn) was explored by measuring the UV–Vis absorption spectrum of the reaction system with a multifunctional microplate detector. Firstly, 1 mg/mL of MIL-53(Fe/Mn) was prepared and sonicated for 20 min to make MIL-53(Fe/Mn), dispersed uniformly without precipitation. Appropriate amounts of MIL-53(Fe/Mn) solution (1 mg/mL), acetic acid–sodium acetate buffer solution (0.2 M, pH 3.0), and TMB (20 mM) were reacted for 20 min at room temperature, and the UV–Vis spectra were measured by a multifunctional microplate detector. The stability of MIL-53(Fe/Mn) was obtained by comparing the catalytic activity of MIL-53(Fe/Mn) against TMB after storage for different durations. The degree of influence of each influencing factor on the simulated enzyme activity was determined by comparing the relative activity (Equation (1a)). The above measurements were repeated three times in parallel to ensure the reliability of the data.

(1a) $$\mathrm{Relative}\;\mathrm{stability}=\frac{A_{n}}{A_{0}}\times 100\%$$

where A is the absorbance and n is the number of days in storage.

### 2.5. The Steady-State Kinetic Analysis

The apparent kinetic constants for MIL-53(Fe/Mn) and MIL-53(Fe) were obtained from the Michaelis–Menten equation (Equation (1b)):

(1b) $$\frac{1}{v}=\frac{K_{m}+\left[S\right]}{V_{\max}\times \left[S\right]}$$

where K_m_ is the Michaelis–Menten constant; v represents the initial reaction speed; V_max_ is the maximum reaction speed; S represents the substrate concentration; A is a certain concentration of MIL-53(Fe/Mn) reacted with different concentrations of TMB, and the absorbance at 652 nm was measured in real time using a multifunctional microplate detector. The actual working concentration of TMB was 0.1, 0.2, 0.3, 0.4, 0.5, 0.6, 0.7, 0.8, 0.9, 1.0 mM, and the total volume of the reaction solution was 200 μL. The initial reaction rates of different concentrations of TMB were calculated according to the changes in absorbance. And then, the apparent kinetic constants V_max_ and K_m_ of MIL-53(Fe/Mn) were calculated according to the fitted Michaelis–Menten curves and double inverse plots. The same method was used to measure the V_max_ and K_m_ values of MIL-53(Fe).

### 2.6. Colorimetric Detection of SO_3_^2−^

To confirm the feasibility of the developed method for SO_3_^2−^ detection, 100 μL of MIL-53(Fe/Mn) solution (1 mg/mL), 100 μL of 20 mM TMB solution, and 1500 μL of acetate buffer solution (0.2 M, pH = 3.0) were first added sequentially and incubated for 20 min at room temperature. Then, 100 μL of the reaction solution was mixed with 100 μL of different concentrations of Na_2_SO_3_ aqueous solution in a 96-well plate. After waiting for 15 min, the UV–visible absorption spectra of the mixed solutions were measured by a multifunctional microplate detector, and the change in absorbance at 300–800 nm was finally observed.

The immunity of a sensing system is a key factor for an analytical method. Therefore, some common cations (K^+^, Na^+^, Mg^2+^, Ga^2+^, Cu^2+^, Zn^2+^, Pb^2+^, Fe^2+^, Co^2+^, Ni^2+^, Cr^2+^, Al^3+^, Fe^3+^), anions (PO_4_^3−^, HPO_4_^2−^, H_2_PO_4_^−^, HCO_3_^−^, SO_4_^2−^, NO_3_^−^, F^−^, CO_3_^2−^, Br^−^, Cl^−^), and some other common substances (CA, TA, VC, GSH, Glu) were investigated to evaluate the selectivity of the developed sensor. The same operation as above was carried out using the same concentration of the potential interfering substances instead of SO_3_^2−^ and then the absorbance values at 652 nm were recorded and compared.

### 2.7. Detection of SO_3_^2−^ in Actual Samples

A representative sample for actual detection, white wine (purchased at a large local supermarket chain), was used for the SO_3_^2−^ test and the results were used to assess the performance of the method in this study. The real samples were prepared as follows: firstly, 1 mL of white wine was diluted 30 times in 50 mL centrifuge tubes with oxygen-free water and mixed slowly. Then, the solution was filtered using an ion chromatography pretreatment column with a specification of 1200 mg/2.5 CC and the filtered solution was stored in a sealed and light-proof container for future use. The SO_3_^2−^ concentration in food samples was measured by using the MIL-53(Fe/Mn)-based colorimetric sensor and ion chromatograph.

## 3. Results and Discussion

### 3.1. Synthesis and Characterization of Bimetallic Nanozyme MIL-53(Fe/Mn)

**Figure 1:**
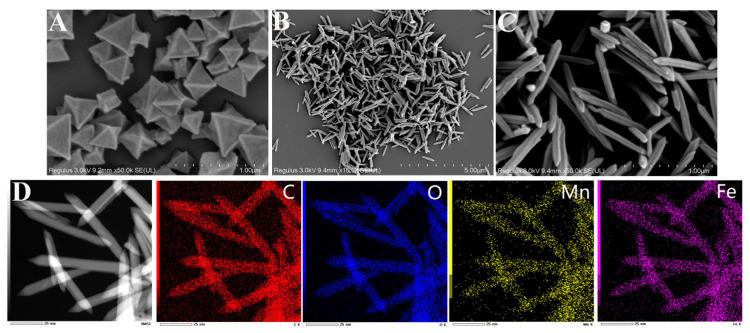
SEM images of (**A**) MIL-53(Fe) (at the magnification of 50k), (**B**,**C**) MIL-53(Fe/Mn) (at the magnification of 10k and 50k). (**D**) Transmission electron microscopy image and elemental mapping of MIL-53(Fe/Mn).

The microscopic morphology and size of the prepared nanozyme are shown in Figure 1. Figure 1A clearly displays that MIL-53(Fe) had an ortho-octahedral cubic structure, which was consistent with previous reports [58]. The MIL-53(Fe/Mn) possessed a three-dimensional structure of an elongated spindle after replacing some Fe active sites by the doping of Mn, with an average particle length of 500 nm and a width of 150 nm (Figure 1B,C). Moreover, the introduction of Mn made the MIL-53(Fe/Mn) significantly different from the pure MIL-53(Fe), indicating that the addition of Mn promoted axial growth. It can also be clearly seen that MIL-53(Fe/Mn) had good dispersibility, which can effectively avoid the aggregation phenomenon in the solution, thereby promoting the adsorption of TMB by MIL-53(Fe/Mn) and benefiting the improvement of the catalytic ability of MIL-53(Fe/Mn). Furthermore, the TEM images and the elemental mapping diagram (Figure 1D) exhibited that the elements Fe, Mn, C, and O of MIL-53(Fe/Mn) distributed uniformly.

**Figure 2:**
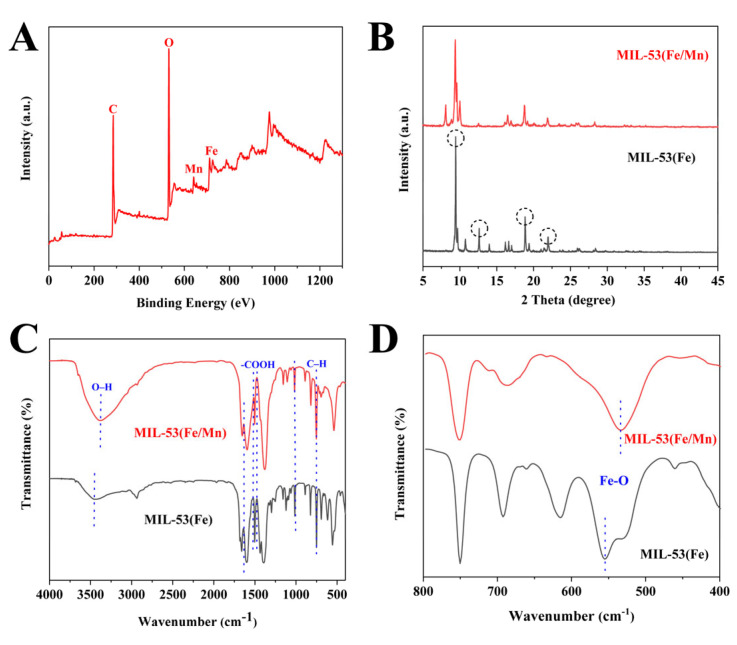
Spectral characterization of MIL-53(Fe) and MIL-53(Fe/Mn). (**A**) The complete XPS spectrum of MIL-53(Fe/Mn). (**B**) The XRD pattern, (**C**) complete FT−IR spectra, and (**D**) local FT−IR spectra of MIL-53(Fe) and MIL-53(Fe/Mn).

The complete XPS spectrum in Figure 2A further confirmed the element composition of MIL-53(Fe/Mn) and demonstrated the successful preparation of MIL-53(Fe/Mn). The XRD spectrum (Figure 2B) presented the crystal structure information of MIL-53(Fe/Mn) and MIL-53(Fe), which showed that MIL-53(Fe) had typical diffraction peaks that were highly consistent with those reported by previous literature [59]. With the introduction of Mn, the crystalline strength of MIL-53(Fe/Mn) had been improved, showing that MIL-53(Fe/Mn) possessed better stability. This is also an important factor that enables MIL-53(Fe/Mn) to maintain good catalytic activity in unfavorable environments. To conduct an in-depth analysis of surface functional groups and molecular structure of nanomaterials, we measured the infrared spectra of MIL-53(Fe) and MIL-53(Fe/Mn), as displayed in Figure 2C. It showed that the materials exhibited obvious spectral peaks at about 3430, 1660, 1590, 1380, 1015, and 750 cm^−1^. The peak at 3430 cm^−1^ was associated with the stretching vibration of the O–H. And the strong peak at 1380 cm^−1^ corresponded to the symmetric vibration of –COOH, indicating that H_2_BDC existed as an organic ligand within the framework of the material. Furthermore, the peak at 750 cm^−1^ was attributed to the bending vibration of C–H in benzene and the vibration of the carboxylate groups. The local magnification spectrum is displayed in Figure 2D. It shows that MIL-53(Fe) had an absorption peak at 555 cm^−1^ due to the presence of the Fe-O node and stretching vibrations, while MIL-53(Fe/Mn) had a lower absorption peak at 537 cm^−1^ due to a shift in the position of the peak introduced by Mn. All the above results illustrate that MIL-53(Fe/Mn) had been successfully prepared.

### 3.2. Oxidase-like Properties of Bimetallic Nanozyme MIL-53(Fe/Mn)

**Figure 3:**
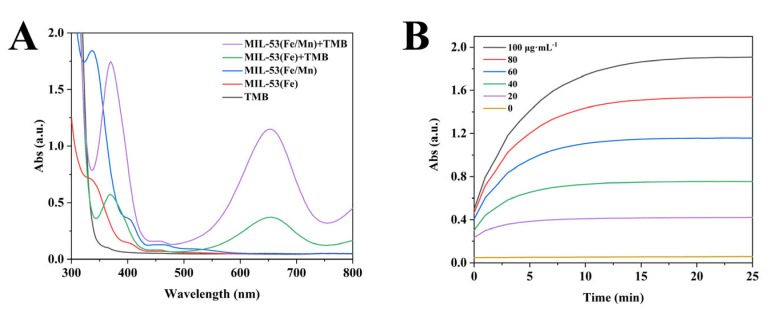
(**A**) UV–Vis absorption spectra of reaction solutions containing different materials; (**B**) plot of absorbance versus time for the TMB reaction solutions catalyzed by bimetallic nanozyme MIL-53(Fe/Mn) with different concentrations.

The most fundamental MIL-53(Fe) has been shown to have oxidase-like activity and, in order to compare the catalytic properties of both MIL-53(Fe/Mn) and MIL-53(Fe), a common chromogenic substrate, TMB, was chosen here as a visualization reagent for the catalytic reaction. Under the catalytic action of the nanozymes, TMB was oxidized to blue oxTMB, making the reaction system appear blue to the naked eye. Generally speaking, under the same conditions, the intensity of a reaction system depends more on the quality of the material’s catalytic performance, which can be clearly compared by measuring the intensity of the absorption peak. As displayed in Figure 3A, there was no obvious characteristic peak at 652 nm when TMB, MIL-53(Fe), and MIL-53(Fe/Mn) existed separately. In contrast, the reaction system of MIL-53(Fe/Mn) and TMB together exhibited a significant absorption peak. Crucially, MIL-53(Fe/Mn)-TMB had a higher characteristic absorption peak compared to MIL-53(Fe)-TMB, indicating that MIL-53(Fe/Mn) possessed a more superior enzyme-like activity than MIL-53(Fe). Furthermore, Figure 3B shows the effect of the concentration of MIL-53(Fe/Mn) on the catalytic ability of the reaction system. As the concentration of MIL-53(Fe/Mn) increased, the absorption peak of the reaction solution at 652 nm gradually increased. With the consumption of the reaction substrate, the reaction remained relatively stable when it proceeded up to 20 min and the absorbance no longer changed.

**Figure 4:**
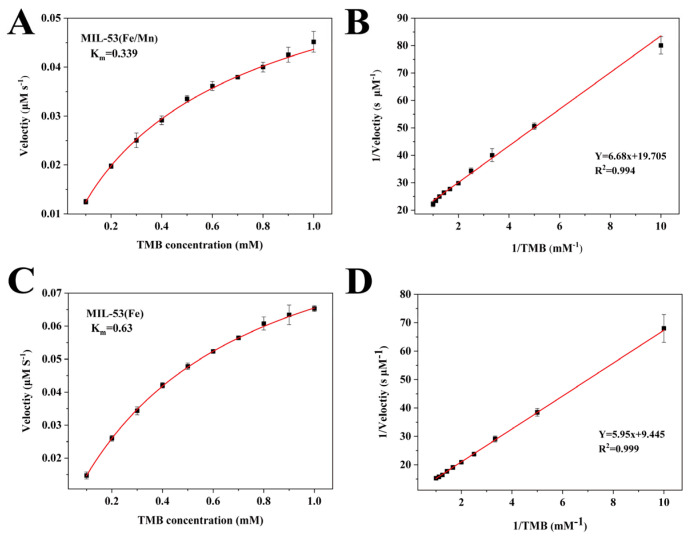
Steady-state kinetic analysis of MIL-53(Fe/Mn) and MIL-53(Fe). (**A**) Kinetic curves of MIL-53(Fe/Mn) and (**C**) MIL-53(Fe), where the actual TMB concentration is 0.1–1.0 mM. (**B**) and (**D**) are double inverted plots calculated from (**A**) and (**C**), respectively (*n* = 3).

In the further investigation and comparison of the catalytic performance of MIL-53(Fe) and MIL-53(Fe/Mn), as shown in Figure 4, MIL-53(Fe) and MIL-53(Fe/Mn) catalyzed the reaction of TMB in full compliance with the Michaelis–Menten equation. From the Michaelis–Menten kinetic curves and the Lineweaver–Burk double inverse plot, V_m_ and K_m_ were calculated. K_m_ represents the Michaelis constant, which is a characteristic physical quantity of enzymes, and a smaller K_m_ value means a stronger affinity between the enzyme and substrate. Therefore, it is clear that the catalytic activity of MIL-53(Fe/Mn) can be determined to be superior by comparing the K_m_ value. All the above evidence confirms that MIL-53(Fe/Mn) manifests better oxidase-like mimetic activity than MIL-53(Fe).

### 3.3. Catalytic Mechanism of Bimetallic Nanozyme MIL-53(Fe/Mn)

**Figure 5:**
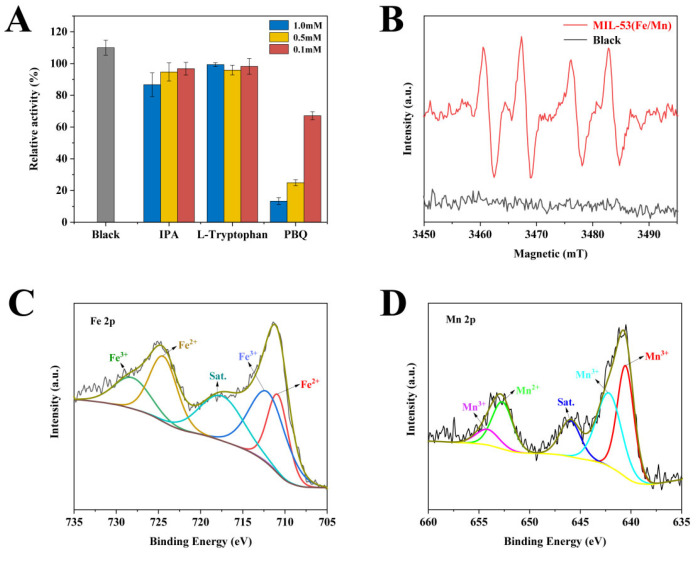
Exploration of the catalytic mechanism of MIL-53(Fe/Mn). (**A**) Reactive oxygen radical−scavenging experiments, (**B**) ESR spectra of O_2_^•−^ capture by DMPO, (**C**) XPS spectra in the Fe 2p region and (**D**) Mn 2p region.

According to previous studies, metal active centers play a key role in the catalytic process of MOF-based nanozymes, which are able to promote the production of reactive oxygen radicals [60,61]. Figure S1 shows the absorbance change in the reaction system under nitrogen blowing and air blowing, and it can be clearly seen that the color absorbance of the solution was greatly reduced after nitrogen discharged the dissolved oxygen in the solution. Thus, the dissolved oxygen in the solution has an essential function in the catalytic process. To further investigate the mechanism of MIL-53(Fe/Mn)-catalyzed TMB, the reactive oxygen radicals generated were first determined by reactive oxygen radical-scavenging experiments. IPA, L-tryptophan, and PBQ are common hydroxyl radical (•OH), singlet oxygen (^1^O_2_), and superoxide radical (O_2_^•−^)-trapping agents, respectively. As depicted in Figure 5A, the absorbance of the reaction system exhibited no significant change after the addition of the trapping agent for the IPA and L-tryptophan; in contrast, the experimental group with added PBQ experienced an obvious inhibitory effect, as evidenced by a clear decrease in absorbance, which indicated that a massive amount of O_2_^•−^ was generated during the catalytic process. In addition, the electron spin resonance (ESR) spectrum (Figure 5B) also confirms the presence of O_2_^•−^. Figure 5B shows that a 1:1:1:1 pattern characteristic signal was detected in the MIL-53(Fe/Mn) reaction system, in contrast to the blank control group in which no characteristic signal was generated. In addition, the electrochemical impedance spectrum (Figure S2) demonstrates a comparison of the electron transfer rates of MIL-53(Fe/Mn) and MIL-53(Fe). Briefly, large diameters represent high impedance and low electron transfer rates. Thus, MIL-53(Fe/Mn) can transfer electrons more efficiently to generate massive amounts of O_2_^•−^, thereby significantly improving the catalytic efficiency of MIL-53(Fe/Mn). Moreover, in order to further investigate the underlying reasons for the excellent catalytic activity of MIL-53(Fe/Mn), a detailed XPS analysis was made for MIL-53(Fe/Mn) and MIL-53(Fe). Figure 5C shows the XPS spectrum of the Fe 2p region, and the peaks at 713 eV, 728 eV and 712 eV, 725 eV are associated with Fe^3+^ and Fe^2+^, respectively [62]. The peaks at 641eV, 653 eV and 643 eV, 655 eV in the Mn 2p region (Figure 5D) can be ascribed to Mn^2+^ and Mn^3+^, respectively [63]. Based on XPS analysis, it is clear that MIL-53(Fe/Mn) has two redox pairs (Fe^2+^/Fe^3+^ and Mn^2+^/Mn^3+^), both of which could be able to produce O_2_^•−^. In addition, the two redox pairs also have a synergistic effect, which results in MIL-53(Fe/Mn) having excellent catalytic activity. In summary, during the entire catalytic process, MIL-53(Fe/Mn) catalyzes the decomposition of dissolved oxygen adsorbed on the surface, resulting in the generation of a large amount of O_2_^•−^ that oxidizes TMB.

### 3.4. Mechanism of MIL-53 (Fe/Mn) Nanozyme for SO_3_^2−^ Detection

**Figure 6:**
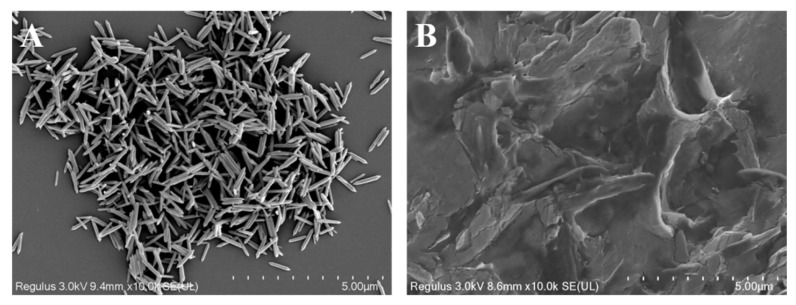
Specific destruction of MIL-53(Fe/Mn) by SO_3_^2−^. (**A**) Before adding SO_3_^2−^, (**B**) after adding SO_3_^2−^.

At present, colorimetric sensors for sulfite detection are mainly based on the strong reduction of sulfites [53,64]. Therefore, the oxidation reaction of SO_3_^2−^ can be judged by measuring the presence of SO_4_^2−^. As shown in Figure S3, the individual MIL-53(Fe/Mn), TMB, SO_3_^2-^, and MIL-53(Fe/Mn)-TMB remained in a clear state in the presence of barium chloride. However, when barium chloride solution was added to the MIL-53(Fe/Mn)-TMB-SO_3_^2-^ mixed solution, a significant turbidity phenomenon occurred due to the large number of BaSO_4_ particles suspended in the solution, indicating the presence of SO_4_^2-^. In order to further investigate the interaction between SO_3_^2−^ and MIL-53(Fe/Mn), the structural changes in MIL-53(Fe/Mn) after the addition of SO_3_^2−^ were explored by scanning electron microscopy. From Figure 6, it can be observed that with the introduction of SO_3_^2−^, the crystal structure of MIL-53(Fe/Mn) was completely and specifically disrupted, resulting in the loss of highly efficient oxidase-like activity. This is mainly attributed to the strong reducing property of SO_3_^2−^ causing the reduction of Mn^3+^ and Fe^3+^ in MIL-53(Fe/Mn) into free Mn^2+^ and Fe^2+^ ions, resulting in the collapse of the crystal structure of MIL-53(Fe/Mn). Building on the previous discussion, the spindle-shaped micro–nanostructures play a crucial role in the oxidase-like activity of MIL-53(Fe/Mn). Therefore, compared to other sensors that rely solely on the reducibility of sulfites, this colorimetric sensor showcases an excellent detection range and limits for sulfites through the combined action of reducibility and specificity, which is worth emphasizing.

In summary, the strong reductive and specific destructive abilities of SO_3_^2−^ lead to a visible reduction in color in the reaction system. In addition, there is an excellent linear relationship between SO_3_^2−^ concentration and absorbance change. These reasons lead to the sensitive and rapid detection of SO_3_^2−^ using the colorimetric sensor. The entire reaction process can be summarized by the following simple reaction equation (Equation (2)):

Fe^2+^ + O_2_ → Fe^3+^ + O_2_^•−^ (2a)

Mn^2+^ + O_2_
→ Mn^3+^ + O_2_^•−^ (2b)

Mn^3+^ + Fe^2+^ → Mn^2+^ + Fe^3^ (2c)

O_2_^•−^ + TMB → oxTMB (blue) (2d)

oxTMB + SO_3_^2−^ →SO_4_^2−^ +TMB (colorless) (2e)

2SO_3_^2−^ + O_2_^•−^ + 2Mn^3+^ →2SO_4_^2−^ + Mn^2+^ (2f)

2SO_3_^2−^ + O_2_^•−^ + Fe^3+^ → 2SO_4_^2−^ + Fe^2+^ (2g)

### 3.5. Optimization of Detection Parameters

**Figure 7:**
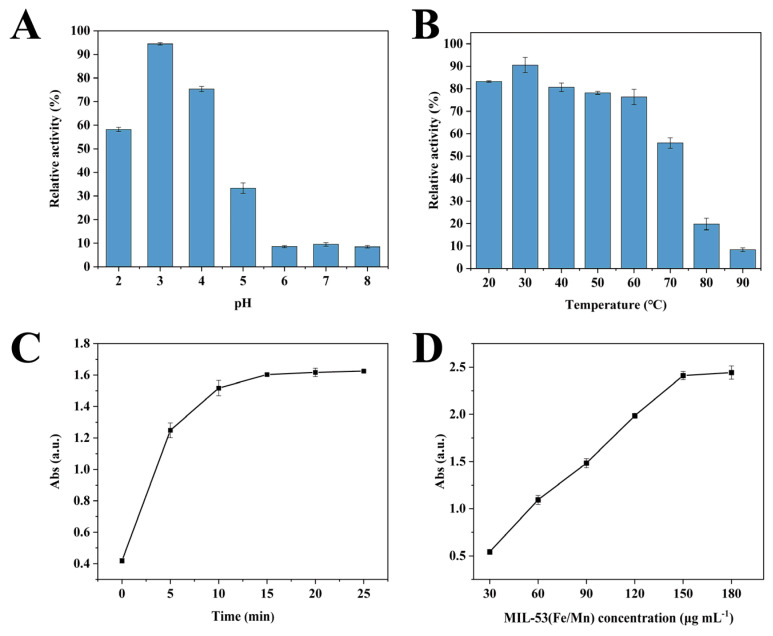
Optimization of experimental conditions. Effect of (**A**) pH, (**B**) temperature, (**C**) reaction incubation time, and (**D**) actual working concentration of MIL-53(Fe/Mn) on the catalytic capacity of the reaction system (*n* = 3).

Natural enzymes are known to be very fragile, and small changes in the external environment can lead to a loss of enzyme activity; nanomimetic enzymes possess similar characteristics. Therefore, finding the optimum operating conditions is essential to maximize the catalytic capacity of the nanomimetic enzymes. Figure 7 shows the extent to which each factor affects the catalytic capacity of the nanoparticle enzyme. MIL-53(Fe/Mn) exhibits good activity between pH 2.0 and 5.0, with the highest enzyme activity at pH 3.0 (Figure 7A). This is mainly attributed to the fact that under acidic conditions, there are more H^+^ in the solution, which is conducive to the amino group in the colorimetric substrate TMB losing electrons and becoming a cation radical. In this state, TMB can easily react with the O_2_^•−^ radicals produced by MIL-53 (Fe/Mn) catalysis to oxidize TMB and form oxTMB. Therefore, alkaline conditions are not conducive to the catalytic oxidation of TMB. However, when the pH is too low, oxTMB will lose another electron and form a stable quinone conjugate monomer structure. This substance appears yellow and has a maximum absorption wavelength at 450 nm, so the absorbance at 652 nm decreases [65]. As depicted in Figure 7B, the catalytic capacity was strongest when the whole reaction system was at 30 °C, after which the enzyme activity decreased as the temperature increased. The catalytic reaction stabilized at 20 min and the color degree of the reaction system no longer changed due to the consumption of substrate (Figure 7C). Therefore, the incubation time for the subsequent experimental reactions was also set at 20 min to ensure complete reaction of the substrate. Additionally, the relationship between MIL-53(Fe/Mn) concentration and the absorbance of the mixed solution can be obtained from Figure 7D. When the MIL-53(Fe/Mn) concentration reached 150 μg mL^−1^, the absorbance started to stabilize. However, in practical research, it was found that an MIL-53(Fe/Mn) concentration of 60 μg mL^−1^ reached the experimental results. Thus, based on the green environmental protection principle and the experimental requirement, a concentration of 60 μg mL^−1^ was selected as the optimal concentration of MIL-53(Fe/Mn).

### 3.6. Colorimetric Detection of SO_3_^2−^

**Figure 8:**
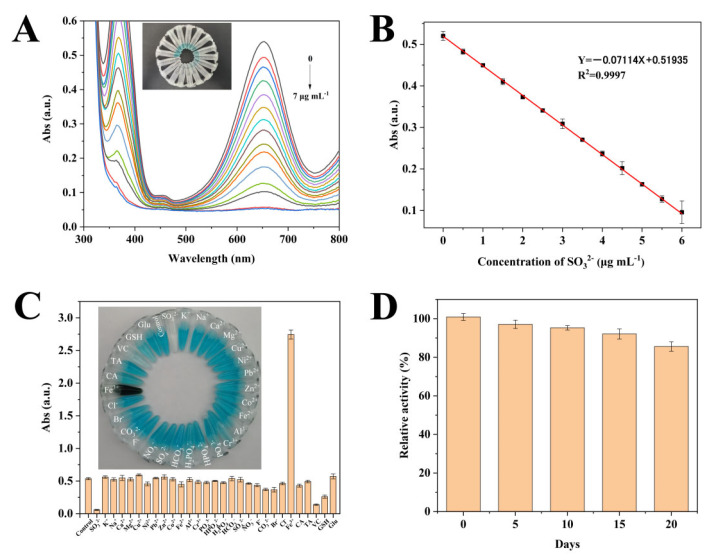
Performance of the colorimetric sensor for the detection of SO_3_^2−^ using the MIL-53(Fe/Mn)−TMB system. (**A**) UV–Vis spectra of the MIL-53(Fe/Mn)−TMB catalytic system corresponding to different concentrations of sulfites (the inset shows the actual inhibition image) and (**B**) the corresponding standard curve. (**C**) Effect of the addition of different anti-interference substances on the catalytic system. (**D**) The relationship between MIL-53(Fe/Mn) placement time and catalytic activity (*n* = 3).

As explored in the previous context, the oxygen dissolved in solution was catalytically decomposed by MIL−-53(Fe/Mn) to convert to O_2_^•−^, and then O_2_^•−^ oxidized the TMB adsorbed on the outer of MIL-53(Fe/Mn) to oxTMB with a blue color, resulting in the color of the solution changing from colorless to blue. Nevertheless, additional SO_3_^2−^ can reduce oxTMB to TMB and convert to SO_4_^2−^ and, in the meantime, the structure of MIL-53(Fe/Mn) can be specifically destroyed by SO_3_^2−^, leading to a loss of catalytic property. Relying on the above principles, a simple, effective, and rapid method for the detection of SO_3_^2−^ was developed. The colorimetric detection of SO_3_^2−^ was carried out using the MIL-53(Fe/Mn)−TMB system under optimal conditions. Afterwards, the inhibitory effects of SO_3_^2−^ were determined by measuring UV absorption spectra in the range of 300–800 nm after adding varying concentrations of SO_3_^2−^ to the MIL-53(Fe/Mn)−TMB system. As shown in Figure 8A, the characteristic UV absorption peak at 652 nm decreased with increasing concentration of SO_3_^2−^, and Figure 8B suggests that there was a good linear relationship between absorbance and SO_3_^2−^ concentration in the range of 0.5–6 μg mL^−1^, which presents the linear relationship of Abs = −0.07114X + 0.51935 (R^2^ = 0.9997). The limit of detection (LOD) was calculated as 0.05 μg mL^−1^ based on the principle of 3 σ/k (σ is the standard deviation of the three blank samples and k is the absolute value of the slope of the standard curve), which showed better detection results than other methods for detecting SO_3_^2−^ (Table 1).

### 3.7. Anti-Interference Capability and Stability

The anti-interference property was investigated by measuring the changes in absorbance at 652 nm with the addition of different interfering substances, including PO_4_^3−^, H_2_PO_4_^−^, HPO_4_^2−^, HCO_3_^2−^, CH_3_COO^−^, SO_4_^2−^, NO_3_^−^, F^−^, CO_3_^2−^, Br^−^, Cl^−^, K^+^, Na^+^, Zn^2+^, Mg^2+^, Cu^2+^, Al^3+^, Fe^3+^, Pb^2+^, Cr^3+^, Vc, GSH, CA, TA, etc. Except for Fe^3+^, VC, and GSH which maintain the same concentration as SO_3_^2−^, the remaining interfering substances were at a concentration 100 times higher. The results are shown in Figure 8C, in which the absorbance of VC and GSH decrease to a certain extent due to the strong reducibility. However, it is worth emphasizing that VC and GSH are rarely used in white wine, so the potential impact can be ignored in practical sample testing. With the strong oxidative power of Fe^3+^, the mixed solution with Fe^3+^ presented a significant color darkening compared to other control groups, which caused serious interference in the detection of SO_3_^2−^. Therefore, the application of the colorimetric sensor is limited when there is a high concentration of Fe^3+^ in the detection matrix. The absorbance of other reaction systems with anti-interference substances was similar to that of the blank control group, indicating that the coexistence of these interference components with SO_3_^2−^ did not affect the detection ability of the colorimetric sensor. Furthermore, the stability of MIL-53(Fe/Mn) was also obtained by comparing the relative activity in different periods (Figure 8D). After 20 days of storage at room temperature, MIL-53(Fe/Mn) still manifested 85% relative activity, indicating that MIL-53(Fe/Mn) possesses phenomenal stability. At the same time, it can be seen from Figure S4 that different batches of MIL-53(Fe/Mn) also exhibit similar catalytic activities, indicating that the synthesis of MIL-53(Fe/Mn) has good reproducibility.

### 3.8. Applicability in Food Samples

In order to confirm the feasibility of MIL-53(Fe/Mn) for the detection of SO_3_^2−^ in food, white wine, as a representative, was selected as the detection object. All tests were repeated three times in parallel under optimized conditions, and the test results are summarized in Table 2. The recoveries of SO_3_^2−^ were obtained from 92.25 to 104.84 with relative standard deviation (RSD) ranging from 0.53 to 5.62, confirming that MIL-53(Fe/Mn) had the potential to be applied in actual food samples. More importantly, the introduction of ion chromatography (IC) ensured the confidence in the detection results of this method.

### 3.9. Smartphone-Based Colorimetric Platform for SO_3_^2−^ Detection

**Figure 9:**
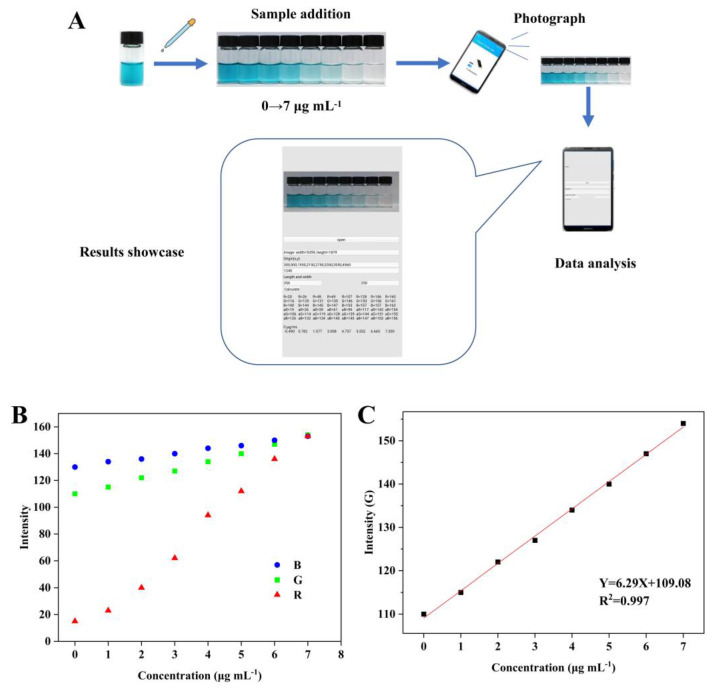
Schematic diagram of a sensing platform for sulfite detection based on a smartphone color recognition application. (**A**) Brief operation process. (**B**) Variation curve of RGB values with sulfite concentration. (**C**) Linear relationship between G value and sulfite concentration.

As the reaction solution appeared to have different degrees of blue depending on the sulfite concentration, a strategy combining a smartphone and a color recognition application was developed to further enable the quantitative detection of SO_3_^2−^ in situ (Figure 9A). Initially, the colors of the reaction system were photographed by a smartphone after adding standard concentrations of SO_3_^2−^ under normal lighting conditions. Then, the average R, G, and B values of the selected region were analyzed, and the average values can maximize the accuracy of the obtained data and avoid errors. Finally, the obtained G values were established with the SO_3_^2−^ concentration (Figure 9B,C). When the concentration of SO_3_^2−^ was 1–7 μg mL^−1^, the linear equation was Y = 6.29X + 109.08 (R^2^ = 0.997), and the detection limit was as low as 0.2 μg mL^−1^. For practical testing in the field, the sample was simply diluted and filtered and then added to MIL-53(Fe/Mn)-TMB acetate buffer solution for 20 min, after which the concentration in the sample was obtained by taking a picture and analyzing with the application. Table S1 shows the detection results of the actual sample using this method, which illustrate that the constructed smartphone colorimetric sensor had good detection performance and can realize the accurate, rapid, and quantitative detection of SO_3_^2−^ in white wine without the need for large equipment.

## 4. Conclusions

In conclusion, bimetallic MIL-53(Fe/Mn) nanozyme was successfully synthesized with efficient oxidase-like activity by a simple one-step hydrothermal reaction. On the basis of the oxidase mimic activity of the prepared MIL-53(Fe/Mn), a rapid, sensitive, and efficient colorimetric sensor for detection of SO_3_^2−^ was developed. The detection mechanism was ascribed to two aspects: on one hand, the nanocrystal structure of MIL-53(Fe/Mn) can be specifically destroyed by SO_3_^2−^, resulting in the loss of catalytic capacity and, on the other hand, SO_3_^2−^ possesses strong reductive capacity, which together lead to a significant reduction in the absorbance of the reaction system. To improve the detection platform for practical application, a smartphone was further applied as a color collector with a simple analysis application. Furthermore, the proposed sensor was successfully applied to actual food samples with excellent performance and good feasibility as expected. This work not only explored the potential application of MIL-53(Fe/Mn) nanozyme in food analysis but also gave a new analytical detection and discrimination method for SO_3_^2−^ detection.

## Figures and Tables

**Table 1:** Comparison of different detection methods with this method.

Method	Material	Linear Range (μg mL^−1^)	LOD (μg mL^−1^)	Ref.
Colorimetric	MIL-53(Fe/Mn)	0.5–6	0.05	This work
Colorimetric	PS-MnO_2_	0–20	0.8	[66]
Colorimetric	Probe 1	0–24	0.1392	[67]
Electrochemistry	Ce^3+^-doped CuO	0.048–32	0.0064	[54]
Electrochemistry	SPCE MWCNT-COOH	0.4–64	1.32	[68]
Electrochemistry	CuNa_2_(OH)_4_	0.4–120	0.1136	[69]
Electrochemistry	CuO-NS	4–128	1.688	[70]
Fluorescence	Probe SPH	0–6.4	0.0184	[71]
Fluorescence	Probe PI	0–8	0.0456	[72]
Fluorescence	BODIPY-Le	0–160	4.64	[73]
Fluorescence	Porous g-C_3_N_4_ Nanosheets	0.16–12	0.024	[74]

**Table 2:** Real sample detection results by the colorimetric sensor.

Sample	IC (mg L^−1^)	Added (mg L^−1^)	Detected by Colorimetric (mg L^−1^)	Recovery (%)	RSD (%, *n* = 3)
White wines	40.22	0	42.17	104.84	0.97
		20	57.52	95.52	5.62
		40	74.00	92.25	1.62
		60	97.35	97.14	0.53

## Data Availability

All related data and methods are presented in this paper. Additional inquiries should be addressed to the corresponding author.

## Supplementary Materials

The following supporting information can be downloaded at: https://www.mdpi.com/article/10.3390/foods12193581/s1, Figure S1: UV–visible absorption spectra of reaction systems under different conditions. Air blowing (black curve) and N_2_ blowing (red curve); Figure S2: The Nyquist curve of MIL-53(Fe/Mn) and MIL-53(Fe); Figure S3: Adding barium chloride solution to acetate buffer solutions containing different solutes. (**A**): MIL-53(Fe/Mn) + BaCl_2_; (**B**): TMB+ BaCl_2_; (**C**): SO_3_^2−^+ BaCl_2_; (**D**): MIL-53(Fe/Mn)−TMB+ BaCl_2_; (**E**): MIL-53(Fe/Mn)-TMB- SO_3_^2−^+ BaCl_2_; Figure S4: The oxidase-like activity of five different batches MIL-53(Fe/Mn); Table S1: Real samples detection results by smartphone-based colorimetric detection platform.
