# Supplementary material for: Rapid Trace Detection of Sulfite Residue in White Wine Using a Multichannel Colorimetric Nanozyme Sensor
Source: Foods. 2023 Sep 26;12(19):3581. doi: 10.3390/foods12193581 (PMC10572540; doi:10.3390/foods12193581)
Supplement: Supplementary file 1 [file foods-12-03581-s001.zip › foods-2586050-supplementary.pdf]

Rapid Trace Detection of Sulfite Residue in White Wine Using A Multichannel Colorimetric Nanozyme Sensor

Xiaoyue Yue[1, 2,3], Long Fu[1],Chaoyun Wu[1], Sheng Xu[4] and Yanhong Bai[1,2,3*]

[1] College of Food and Bioengineering, Zhengzhou University of Light Industry, Zhengzhou 450001, China; 2017059@zzuli.edu.cn

[2] Key Laboratory of Cold Chain Food Processing and Safety Control (Zhengzhou University of Light Industry), Ministry of Education

[3] Henan Key Laboratory of Cold Chain Food Quality and Safety Control

[4] College of Computer and Communication Engineering, Zhengzhou University of Light Industry, Zhengzhou 450001, China; xusheng@zzuli.edu.cn

* Correspondence: baiyanhong212@163.com (Y.B.)

***Corresponding Author: Yanhong Bai**

E-mail: baiyanhong212@163.com

Captions of Figures

Figure S1. UV-visible absorption spectra of reaction systems under different conditions. Air blowing (Black curve) and N_2 blowing (Red curve)

Figure S2. The nyquist curve of MIL-53(Fe/Mn) and MIL-53(Fe)

Figure S3. Adding barium chloride solution to acetate buffer solutions containing different solutes. A: MIL-53(Fe/Mn)+$BaCl_2$; B: TMB+ $BaCl_2$; C: SO_3^{2-}+ $BaCl_2$; D: MIL-53(Fe/Mn)-TMB+ $BaCl_2$; E: MIL-53(Fe/Mn)-TMB- SO_3^{2-}+ $BaCl_2$;

Figure S4. The oxidase-like activity of five different batches MIL-53(Fe/Mn)

Table S1. Real samples detection results by smartphone-based colorimetric detection platform

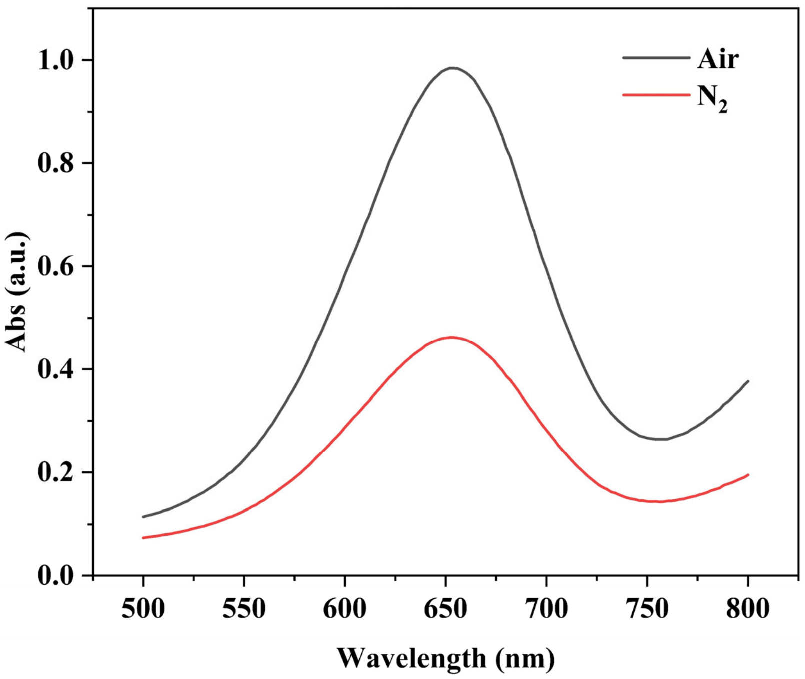

Figure S1. UV-visible absorption spectra of reaction systems under different conditions. Air blowing (Black curve) and N_2 blowing (Red curve).

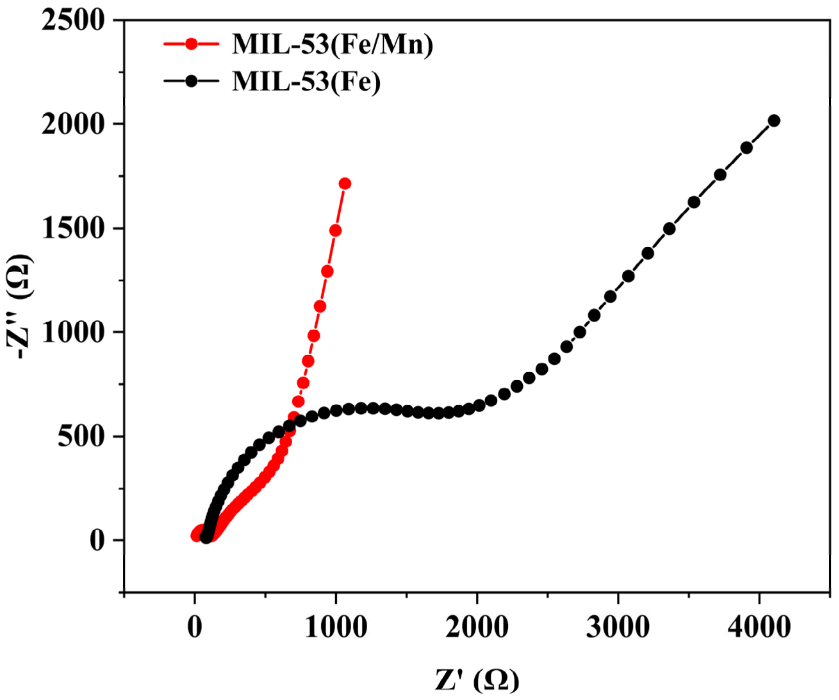

Figure S2. The nyquist curve of MIL-53(Fe/Mn) and MIL-53(Fe).

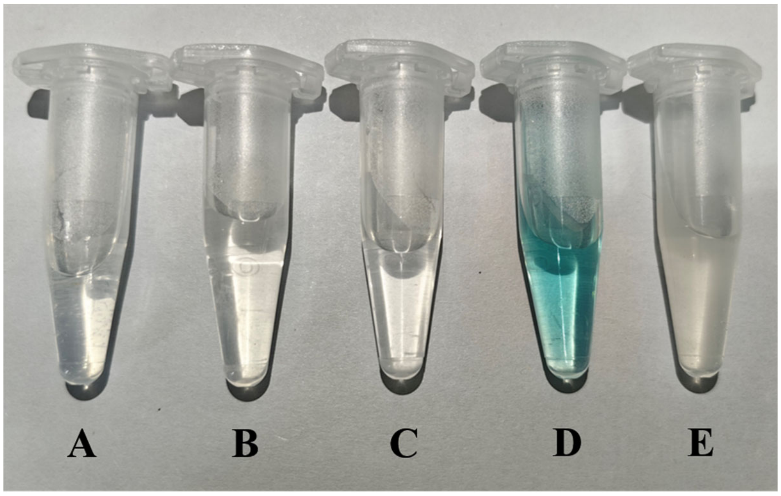

Figure S3. Adding barium chloride solution to acetate buffer solutions containing different solutes. A：MIL-53(Fe/Mn)+$BaCl_2$; B: TMB+ $BaCl_2$; C: SO_3^{2-}+ $BaCl_2$; D: MIL-53(Fe/Mn)-TMB+ $BaCl_2$; E: MIL-53(Fe/Mn)-TMB-SO_3^{2-}+ $BaCl_2$.

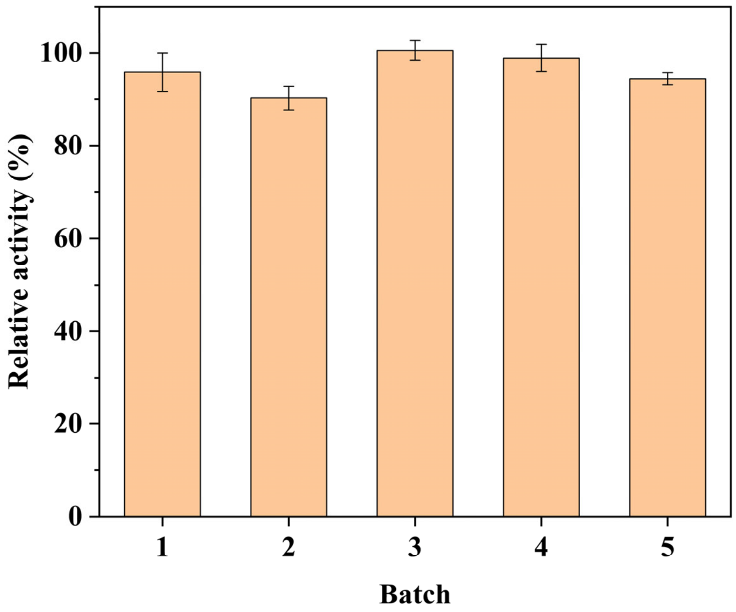

Figure S4. The oxidase-like activity of five different batches MIL-53(Fe/Mn).

Table S1. Real samples detection results by smartphone-based colorimetric detection platform.

Sample	IC ($mg\ L^{-1}$)	Add ($mg\ L^{-1}$)	Detected by smartphone ($mg\ L^{-1}$)	Recovery (%)	RSD (%, n = 3)
White wines	40.22	0	40.95	101.82	17.7
		20	60.03	99.68	6.0
		40	82.29	102.58	6.7
		60	98.19	97.97	5.6
